# Supplementary material for: The Systems Biology Research Tool: evolvable open-source software
Source: BMC Syst Biol. 2008 Jun 29;2:55. doi: 10.1186/1752-0509-2-55 (PMC2446383; doi:10.1186/1752-0509-2-55)
Supplement: Additional file 1 — SBRT Archive. An archive of the current version of the Systems Biology Research Tool. [file 1752-0509-2-55-S1.zip › sbrt-1.4.0/doc/developers_guide/api/sbrt/shell/io/AbstractSbrtFile.html]

AbstractSbrtFile


|  |  |  |
| --- | --- | --- |
| **Overview** \| **Package** \| **Class** \| **Use** \| **Tree** \| **Deprecated** \| **Index** \| **Help** | |  |
| **PREV CLASS**   **NEXT CLASS** | **FRAMES**    **NO FRAMES**     **All Classes** |  |
| SUMMARY: NESTED \| FIELD \| CONSTR \| METHOD | DETAIL: FIELD \| CONSTR \| METHOD |  |


---


## sbrt.shell.io Class AbstractSbrtFile

```
java.lang.Object
  sbrt.shell.io.AbstractSbrtFile
```

---

``` public class AbstractSbrtFile extends java.lang.Object ```

This class provides a helper method for SBRT files.

**Author:**
:   This class was written and documented by Jeremiah Wright while in the
    Wagner lab.

---

**Constructor Summary**

`AbstractSbrtFile()`


| **Method Summary** | |
| --- | --- |
| `static java.lang.String` | `getCanonicalName(java.lang.String fileName)`             Returns the canonical name of the specified file. |

**Methods inherited from class java.lang.Object**

`clone, equals, finalize, getClass, hashCode, notify, notifyAll, toString, wait, wait, wait`

**Constructor Detail**

### AbstractSbrtFile

```
public AbstractSbrtFile()
```


**Method Detail**

### getCanonicalName

```
public static java.lang.String getCanonicalName(java.lang.String fileName)
```

:   Returns the canonical name of the specified file.

    :   **Parameters:**: `fileName` - the name of the file. **Returns:**: the canonical name of the specified file. **See Also:**: `File.getCanonicalPath()`


---


|  |  |  |
| --- | --- | --- |
| **Overview** \| **Package** \| **Class** \| **Use** \| **Tree** \| **Deprecated** \| **Index** \| **Help** | |  |
| **PREV CLASS**   **NEXT CLASS** | **FRAMES**    **NO FRAMES**     **All Classes** |  |
| SUMMARY: NESTED \| FIELD \| CONSTR \| METHOD | DETAIL: FIELD \| CONSTR \| METHOD |  |


---
